# Peritoneal reflection involvement as a prognostic factor in rectal cancer. Long-term oncological outcomes from a prospective study

**DOI:** 10.1007/s00384-025-04909-7

**Published:** 2025-05-10

**Authors:** Eduardo Alvarez-Sarrado, Matteo Frasson, Jorge Sancho-Muriel, Maria Jose Gomez-Jurado, Hanna Cholewa, Vicent Primo-Romaguera, Monica Millan, Adela Batista, Polina Rudenko, Blas Flor-Lorente, Eduardo Garcia-Granero, Francisco Giner

**Affiliations:** 1 Department of Colorectal Surgery, University and Polytechnic Hospital La Fe, Av. Fernando Abril Martorell 106, 46026 Valencia, Spain; 2 Department of Surgery, University of Valencia, Valencia, Spain; 3 Abdominal Imaging, Department of Radiology, University and Polytechnic Hospital La Fe, Valencia, Spain; 4 Pathology Department, University of Valencia, Valencia, Spain; 5 Pathology Department, University and Polytechnic Hospital La Fe, Av. de Blasco Ibáñez, 13, 46010 València, Spain

**Keywords:** Rectal cancer, Peritoneal reflection, Peritoneal involvement, Local recurrence, Carcinomatosis

## Abstract

**Purpose:**

To assess the relevance of peritoneal reflection involvement in long-term oncological outcomes in patients with rectal cancer.

**Methods:**

Prospective observational study from a specialized colorectal unit that included a consecutive series of patients undergoing mesorectal excision for rectal cancer. Peritoneal reflection (PR) involvement was evaluated on pathological examination using Shepherd’s classification. Overall survival (OS), disease-free survival (DFS), and local recurrence (LR) were assessed.

**Results:**

One hundred sixty patients were included in the present analysis. Peritoneal involvement was present in 28.2% of the 85 tumors above or at the level of PR. There were no differences in OS, DFS, or LR according to tumor’s height location. The 5-year OS, DFS, and LR for tumors involving PR were 58.3%, 61.7%, and 30.3%, respectively. Patients with peritoneal involvement had a higher LR rate (*p* = 0.02) and shorter OS (*p* = 0.04). Shepherd’s grade 4 peritoneal involvement was an independent risk factor for OS (HR 2.9; 95% CI 1.1–9.5, *p* = 0.04) and LR (HR 4.2; 95% CI 1.2–16.9, *p* = 0.04).

**Conclusion:**

After rectal cancer resection, peritoneal involvement is an independent risk factor for local recurrence and poor survival.

## Introduction

Circumferential resection margin (CRM) involvement after surgical resection is widely known to be a risk factor for both local recurrence (LR) and poor survival [1–5]. However, for anterior tumors above the peritoneal reflection (PR), peritoneal involvement should be carefully assessed and independently reported from CRM involvement [6, 7]. Moreover, most publications do not take this aspect into account and data concerning CRM involvement for anterior tumors may be misunderstood [8–10].

Previous publications about oncological outcomes in rectal cancer have mainly focused on pathological CRM (pCRM) status as it has been proved to be one of the most important LR-related factors. Patients with pCRM involvement have a 5-year LR rate around 23.7–26.7% [10, 11]. However, up to 25% of anterior rectal surface is covered by peritoneum, even if this fact is frequently ignored [12, 13]. In upper and middle rectal tumors involving the serosal surface, the concept of CRM is not applicable and they must be classified as pT4a as they are intraperitoneal tumors [6, 7, 14]. Additionally, tumors above or at the level of PR may have peritoneal spread in addition to the classical lymphatic and hematologic patterns of dissemination developed by lower rectal tumors [15].

High-resolution magnetic resonance imaging (MRI) is the gold standard for rectal cancer local staging as it can determine the depth of invasion and distance to anal margin and predict the involvement of mesorectal fascia with high accuracy [5, 16–21]. This information is crucial to select patients for neoadjuvant treatment and to determine the appropriate surgical technique [8, 10, 22, 23]. Nonetheless, peritoneal involvement is not always detected by preoperative MRI, as shown in our previous publication, reaching an overall accuracy of 80.5–95.9% [19, 24]. In 1995, Shepherd established four degrees of peritoneal involvement according to the depth of invasion [25]. They later demonstrated that serosal invasion is a relevant risk factor for LR and poor OS; however, this association did not reach statistical significance in the multivariate analysis [26]. Consequently, certain authors have advocated for neoadjuvant systemic chemotherapy protocols in the management of upper rectal tumors with serosal involvement, mirroring most recent strategies employed for locally advanced colon cancer. This recommendation is supported by studies such as the FOxTROT trial, aiming to induce tumor downstaging and mitigate the potential for peritoneal dissemination [27].

Despite CRM involvement relevance has been widely investigated, very few publications have focused on peritoneal reflection involvement. Recently, several authors have highlighted the importance of MRI accuracy for identification and determination of the level of PR [12, 28–31]. Nonetheless, the prognostic importance of peritoneal involvement on oncological outcomes remains to be determined.

The primary objective of this prospective study is to assess PR involvement as a relevant factor determining local recurrence (LR), disease-free survival (DFS), and overall survival (OS) after rectal resection for adenocarcinoma.

## Methods

This is a prospective, observational study conducted by a specialized multidisciplinary colorectal unit at a tertiary hospital. This manuscript has been written following the STROBE guidelines.

### Ethical statements

The study was approved by the institution’s ethics committee and written informed consent was obtained from each patient. Registry number: 2016/0373.

#### Description of participants

All patients with histopathologically confirmed rectal adenocarcinoma undergoing surgical resection with total or subtotal mesorectal excision were enrolled from June 2016 to May 2019. Some of the patients of the present analysis were already included in a previous publication focused on overall MRI accuracy for PR location and involvement [24].

#### Radiologic assessment

High-resolution MRI was performed in a 1.5-T MRI scanner (General Electric Medical System, Milwaukee, WI, USA) with a pelvic phased-array coil. MRI protocol details are explained in our previous publication [24].

MRI images were evaluated and discussed at the multidisciplinary board. Peritoneal reflection involvement was defined as direct contact or nodular extension of the tumor into the peritoneal surface. CRM involvement was defined as direct contact or tumor within 1 mm (mm) to mesorectal fascia.

#### Pathological assessment

A double-ink technique was applied, Indian ink on mesorectal extraperitoneal surface and orange ink for the peritoneal surface. Detailed pathological protocol and dying technique are explained in our previous paper [24].

**Fig. 1:**
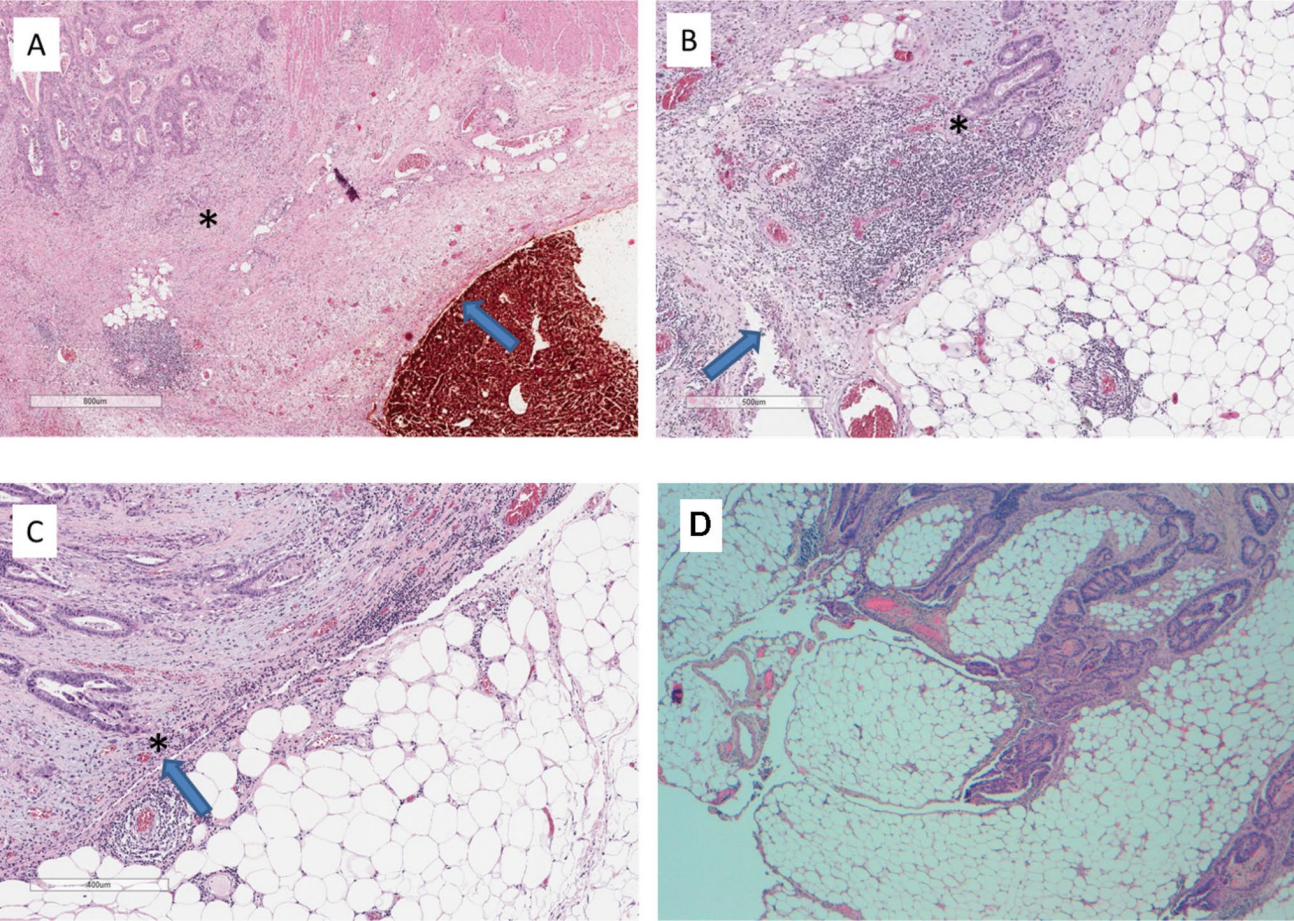
Microscopy photography of formalin-fixed and paraffin-embedded rectal cancer specimens showing peritoneal involvement according to Shepherd’s classification: **A** Shepherd’s grade 1 (HE stain; 2.8 ×, bar = 800 μm); **B** Shepherd’s grade 2 (HE stain; 4.6 ×, bar = 500 μm); **C** Shepherd’s grade 3 (HE stain; 5.8 ×, bar = 400 μm); **D** Shepherd’s grade 4 (HE stain; 4 ×). Asterisk (*) shows tumor cells at invasion front. Arrow shows peritoneal surface with orange dye. HE, hematoxylin–eosin

Tumors located within 5 mm of the peritoneal reflection were considered at the level of PR for the purpose of analysis. Peritoneal involvement was assessed according to Shepherd’s classification into four degrees, as shown in Table 1 [25]. Grades 1–2 were considered free of serosal involvement and grades 3–4 as involved serosa. Figure 1 shows microscopy photographies of pathological findings according to Shepherd’s peritoneal involvement degrees. CRM involvement was defined as a tumor within 1 mm to mesorectal fascia. Both pathological data, PR and pCRM involvement, were considered for survival analysis.

**Table 1:** Pathological characteristics of different peritoneal involvement grades defined by Shepherd

Shepherd’s degrees of peritoneal involvement [25]	
Grade 1	Free of peritoneal involvement
Grade 2	Mesothelial inflammation or hyperplastic with tumor close but not actually present at the peritoneal surface
Grade 3	Microscopic involvement of peritoneal surface
Grade 4	Peritoneal ulceration with free tumor cells in peritoneum

#### Follow-up and oncological outcomes

Recommendations for neoadjuvant treatment were discussed by the multidisciplinary board for all patients. Preoperative long-course chemoradiotherapy (total dose of 45–50.4 Gy with 1.8–2 Gy per fraction) with concomitant capecitabine-based chemotherapy was considered for cT3 and cT4 low and middle rectal cancer with high-risk factors such as involved mesorectal fascia. None of the patients received a total neoadjuvant therapy (TNT) regimen during the study period, as it was conducted prior to the implementation of TNT strategies. Oxaliplatin-based adjuvant regimes were given to selected patients after surgery. None of the patients received postoperative radiotherapy.

Patients were followed by serial clinical examination and carcinoembryonic antigen assessment every 3 months during the first year, every 6 months during the second year, and annually thereafter. Thoracoabdominal computed tomography scanning was performed every 6 months for the first 2 years and annually thereafter for 5 years. Colonoscopy was performed after 1 year and 3 to 5 years thereafter, depending on individual patient risk. If recurrence was suspected, then further diagnostic methods were used as required.

OS was defined as the time from surgery to death for any cause. DFS was defined as the time from surgery to date of local recurrence or distant metastases diagnosis. LR was defined as the presence of tumor at anastomosis, pelvic mass, peritoneal carcinomatosis, or locoregional lymphatic recurrence. Local recurrence-free survival (LRFS) was defined as the time from surgery to date of LR.

#### Statistical analysis

SPSS software (IBM SPSS Statistics for Macintosh, version 24.0, IBM Corp, Armonk, NY) was used for statistical analysis. Categorical variables were compared among groups using *χ*^2^ and Fisher exact tests. Continuous variables were compared by ANOVA or Kruskal–Wallis test. All time-to-event variables were calculated from the date of surgery. The univariate influence of prognostic factors on LR, DFS, and OS was analyzed for all of the groups with the Kaplan–Meier method and the log-rank (Mantel-Cox) test. A Cox multivariate regression model was constructed including variables with *p* < 0.10 at univariate analysis. Proportional hazards assumption of the Cox model was assessed. Statistical significance for all the results was defined as *p* < 0.05.

## Results

### Patient’s baseline

A total of 160 patients were enrolled in the study. The median age was 65 years (interquartile range (IQR): 57–73 years) and 56.3% were males. 27.5% of tumors were located in the upper third (11–15 cm from anal verge), 39.4% in the middle third (7–10 cm), and 33.1% in the lower rectum (0–6 cm). Sixty-four patients (40%) received neoadjuvant chemoradiotherapy.

After surgical resection, good-quality mesorectal excision plane was achieved in 126 (78.8%) specimens. CRM was involved in 11 cases (6.9%). When extended resections for locally advanced pT4b-tumors are excluded, CRM was involved in 6/146 (4.1%).

After pathological examination, 22 (13.8%) tumors were located above the PR, 63 (39.4%) at the level of PR, and 75 (46.9%) below the PR. For tumors located at or above the PR (*n* = 85), peritoneal involvement was confirmed in 24 out of 85 (28.2%) patients, with 14 classified as grade 3 and 10 as grade 4 according to Shepherd’s classification. For patients with PR involvement, TME plane was satisfactory in 17 cases (70.8%), partially satisfactory in 4 (16.6%), and unsatisfactory in 3 (12.6%). Demographic, preoperative, and pathological data for the whole group are shown in Table 2.

**Table 2:** Demographic, preoperative, and pathological characteristics of patients

Data for the whole group (*n* = 160)			
		*N*	%
Patient’s variables	Sex (male/female)	90/70	56.3/43.7
	Median age (IQR)	65 (57–73)	
	Rectal tumor height - Upper (11–15 cm) - Middle (7–10 cm) - Lower (0–6 cm)	44 63 53	27.5 39.4 33.1
	rmN +	87	54.4
	rmCRM +/threatened	55	34.4
	Neoadjuvant chemoradiation	64	40
Surgery	Procedure: - Low anterior resection - Hartmann’s procedure - Abdominoperineal resection - Pelvic exenteration - Other	100 8 35 7 10	62.5 5 21.9 4.4 6.2
	Extended resection	32	20
	Laparoscopic	102	63.7
	Focal carcinomatosis	9	5,6
	Obstruction	5	23.1
	Tumor’s perforation	2	1.2
	RBC transfusion	5	3.1
Pathological exam	Quality of mesorectum - Complete - Nearly complete - Non-complete	126 18 16	78.8 11.2 10
	Poor tumoral differentiation	15	9.4
	Tumoral budding	103	64.4
	Lympho-vascular invasion	53	33.1
	Neural invasion	28	17.5
	T stage: - pT1-2 - pT3 - pT4a - pT4b	67 58 19 16	41.9 36.2 11.9 10
	pN +	62	38.7
	Relationship with peritoneal reflection - Above - At the level - Below	22 63 75	13.8 39.4 46.9
	Shepherd’s degree - Not applicable - 1 - 2 - 3 - 4	75 51 10 14 10	46.9 31.9 6.2 8.8 6.2
	pCRM +	11	6.9

Abbreviations: *IQR*, interquartile range; *rm*, magnetic resonance-preoperative staging; *N* +, positive adenophaties; *CRM* +, circumferential resection margin involvement; *RBC*, red blood cell; *p*, final pathological staging

### Oncological outcomes

The median follow-up time was 67 months. Five-year OS, 5-year DFS, and 5-year LRFS were 75% (95% CI, 68.3–81.6), 72.1% (95% CI, 65–79.1), and 84.4% (95% CI, 78.5–90.2), respectively. Isolated LR occurred in 5 patients (3.1%) whereas 18 (11.2%) patients had both LR and distant metastasis, and 21 patients (13.1%) developed distant metastasis only.

The median time to LR after surgery was 24 months (IQR: 12–39). Patterns of local recurrence included carcinomatosis in 11 patients, pelvic mass and carcinomatosis in 2 patients, and pelvic recurrence alone in 9 patients. Three patients showed locoregional lymph node involvement, one as the sole LR site. In our series, pT4 tumors represent 21.9% of cases, with LR occurring in 28.6% of these cases: 9 patients as carcinomatosis, while 1 patient developed pelvic recurrence. Four patients underwent pelvic exenteration after local recurrence. The median OS after LR diagnosis was 14 months (IQR: 4–22 months).

The median time to diagnosis of distant metastasis after surgery was 15 months (IQR: 6–22). Metastasis patterns were observed as follows: 7 cases presented with hepatic metastases only, 13 cases hepatic and pulmonary metastases, 13 cases pulmonary metastasis only, and 6 cases showed multiple-organ metastases. The median OS following metastatic progression was 22 months (IQR: 8–47).

#### Location of the tumor in relation with the peritoneal reflection

According to tumor’s location, no statistical difference was found in 5-year OS (90% vs. 77.3% vs. 79.5%, *p* = 0.603), 5-year DFS (76.2% vs. 62.1% vs. 72.6%, *p* = 0.58), and LRFS (90.5% vs. 81% vs. 84.1%, *p* = 0.639) for tumors above, at or below the PR, respectively.

#### Involvement of peritoneal reflection

Table 3 shows the baseline characteristics in patients with or without peritoneal involvement. More frequently, patients with peritoneal involvement required an extended resection and had more advanced tumors at pathological staging with higher proportion of lympho-vascular invasion, pT4 stages, and lymph node involvement.

**Table 3:** Preoperative and pathological characteristics in both groups, based on the presence or absence of peritoneal involvement

Demographic’s variables				
		No peritoneal involvement (*n* = 136) *N* (%)	Peritoneal involvement (*n* = 24) *N* (%)	*p*
Patient’s variables	Male sex	76 (55.8)	14 (58.3)	0.82
	rmN +	72 (52.9)	15 (62.5)	0.42
	Neoadjuvant chemoradiation	57 (41.9)	7 (29.1)	0.24
	Laparoscopic approach	91 (66.2)	11 (45.8)	0.07
	Extended resection	23 (16.9)	9 (37.5)	0.02*
	Obstruction	2 1.5)	3 (12.5)	0.004*
	Tumor’s perforation	3 (2.2)	3 (12.5)	0.04*
Pathological data	Complete mesorectum	109 (80.1)	17 (70.8)	0.3
	Poor tumoral differentiation	12 (14.7)	3 (12.5)	0.57
	Tumoral budding	86 (63.2)	17 (70.8)	0.72
	Lympho-vascular invasion	40 (29.4)	13 (54.2)	0.018*
	Neural invasion	21 (15.4)	7 (29.2)	0.1
	pT4	11 (8.1)	24 (100)	< 0.001*
	pN +	46 (33.8)	16 (66.7)	0.002*
	pCRM +	9 (6.6)	2 (8.3)	0.76

^*^Statistically significant *p*-value (*p* < 0.05)

Abbreviations: *rm*, magnetic resonance-preoperative staging; *N* +, positive adenophaties; *p*, final pathological staging; *T4*, T4 staging on TNM classification; *CRM* +, circumferential resection margin involvement

**Fig. 2:**
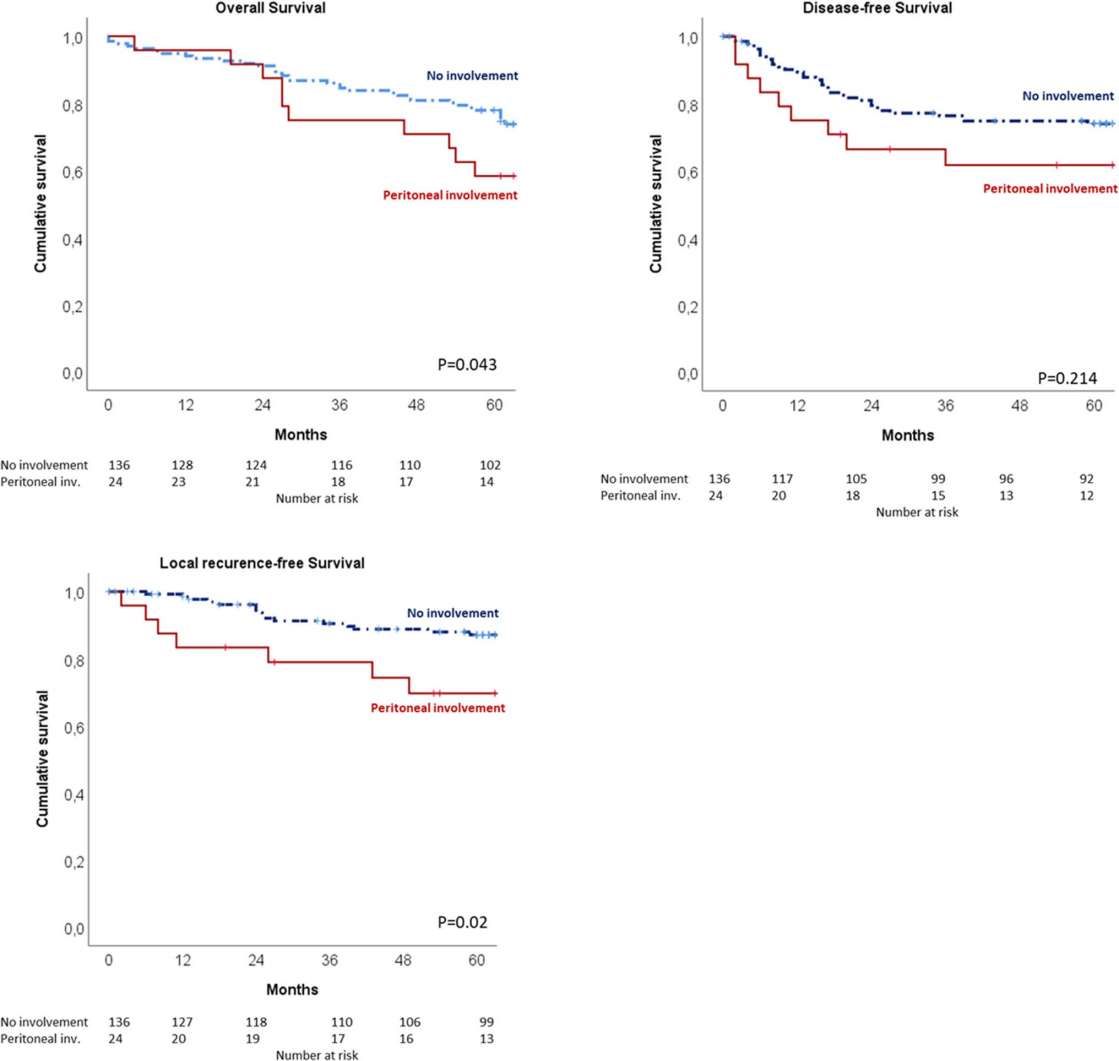
Kaplan–Meier curves for OS, DFS, and LRFS for patients with and without peritoneal involvement

For patients with peritoneal involvement, 5-year OS (58.3% vs. 73.8%, *p* = 0.043) and 5-year LRFS (69.7% vs. 87.1%, *p* = 0.02) were significantly shorter. Patients with Shepherd’s grade 4 peritoneal involvement compared to the rest of the patients had the worse long-term outcomes with 5-year OS (50% vs. 76.7%, *p* = 0.05), 5-year DFS (50% vs. 73.7%, *p* = 0.027), and 5-year LRFS (50% vs. 86.8%, *p* < 0.001). Kaplan–Meier curves for OS, DFS, and LRFS are shown in Fig. 2.

#### Risk factor for OS, DFS, and LR

On multivariate Cox regression analysis, Shepherd’s grade 4 peritoneal involvement was confirmed as an independent prognostic factor for higher LR (HR 4.2, 95% CI 1.2–16.9) and worse OS (HR 2.9, 95% CI 1.1–9.5). Table 4. summarizes the results of univariate and multivariate analysis for OS, DFS, and LRFS.

**Table 4:** Univariate and multivariate Cox analysis for OS, DFS, and LRFS in patients with resected rectal cancer

		Overall survival				Disease-free survival				Local recurrence-free survival			
		Univariate analysis		Multivariate analysis		Univariate analysis		Multivariate analysis		Univariate analysis		Multivariate analysis	
		Hazard ratio	*p*	Adjusted-HR	*p*	Hazard ratio	*p*	Adjusted-HR	*p*	Hazard ratio	*p*	Adjusted-HR	*p*
Variables													
Preoperative	Male sex	1.1 (0.5–2.6)	0.73			1.2 (0.6–2.5)	0.55			1.2 (0.5–3)	0.62		
	Lower vs. upper-middle third	0.9 (0.4–2.3)	0.98			1.1 (0.5–2.3)	0.73			0.9 (0.4–2.3)	0.85		
	Below vs. at/above PR	0.9 (0.4–2.1)	0.88			1.3 (0.6–2.6)	0.46			1.2 (0.5–2.8)	0.72		
	rmT4	1.2 (0.5–3.1)	0.56			1.5 (0.7–3.2)	0.23			2.1 (0.9–2.3)	0.09	1.1 (0.5–2.2)	0.72
	rmCRM+	2 (0.8–4.6)	0.09	1.1 (0.4–2.8)	0.43	2.3 (1.2–4.8)	0.01*	1.4 (0.7–2.7)	0.39	1.9 (0.8–4.7)	0.14		
	rmN+	2.2 (0.9–5.3)	0.07	1.2 (0.5–2.6)	0.67	2.3 (1.1–4.9)	0.02*	1.1 (0.5–2.5)	0.91	1.6 (0.7–4.1)	0.29		
	Neoadjuvant CRT	3.7 (1.5–9)	0.002*	2.9 (1.2–6.6)	0.01*	2.4 (1.2–4.9)	0.01*	1.9 (1.1–3.7)	0.03*	2.2 (0.9–5.3)	0.08	1.1 (0.5–2.4)	0.83
Intraoperative	Extended resection	0.9 (0.3–2.5)	0.83			1 (0.4–2.4)	0.59			2 (0.7–5.2)	0.17		
	Laparoscopic approach	0.8 (0.3–1.8)	0.59			1 (0.5–2.1)	0.91			0.9 (0.4–2.1)	0.75		
	RBC transfusion	3.4 (0.5–21)	0.16			1.7 (0.3–10.7)	0.54			4.2 (0.7–27)	0.09	1.1 (0.5–2.2)	0.78
	Tumoral obstruction	0.8 (0.7–1.1)	0.39			1.3 (0.3–7.3)	0.54			1.2 (0.2–10.7)	0.87	1 (0.5–2)	0.89
	Rectal perforation	2.5 (0.4–14.8)	0.26			0.7 (0.6–1.1)	0.18			0.9 (0.8–1.1)	0.35		
	Focal carcinomatosis	16.5 (1.6–165)	0.002*	4.7 (1.1–21)	0.04*	25 (1.4–475)	0.03*	15.8 (2.2–115)	0.006*	20.4 (2–205)	<0.001*	6.2 (1.3–29.3)	0.02*
Postoperative	TME quality (incomplete)	0.9 (0.3–2.5)	0.89			0.7 (0.3–1.7)	0.53			0.7 (0.3–2)	0.54		
	Poor tumoral differentiation	2.8 (0.9–8.9)	0.07	1.2 (0.4–2.9)	0.45	6.2 (2–19.6)	<0,001*	1.6 (0.7–3.7)	0.31	3.5 (1.1–11.5)	0.02*	1.1 (0.3–4.1)	0.83
	Tumoral budding	2.1 (0.7–6)	0.15			2.2 (0.9–5.2)	0.06	1.1 (0.4–2.3)	0.66	1.8 (0.6–5.3)	0.24		
	Lympho-vascular invasion	2.6 (1.1–6)	0.02*	1.1 (0.4–3.4)	0.78	3.4 (1.6–6.9)	0.001*	1.2 (0.5–3.1)	0.74	3.2 (1.3–7.7)	0.01*	2.7 (0.7–10.7)	0.16
	Neural invasion	2.3 (0.9–6.1)	0.06	1 (0.5–2.2)	0.86	4.7 (2–11.1)	<0,001*	1.9 (0.8–4.6)	0.13	3.4 (1.5–10.5)	0.003*	2.6 (0.7–8.9)	0.13
	pT4	1.6 (0.6–4.1)	0.28			2.4 (1.1–5.2)	0.02*	1.2 (0.5–2.5)	0.73	3.5 (1.4–8.7)	0.007*	2.3 (0.5–10.4)	0.29
	pN+	4 (1.6–9.7)	0.001*	2.5 (1.1–6.3)	0.03*	3.9 (1.9–8.1)	0,001*	2.2 (1.1–4.3)	0.02*	2.9 (1.2–7.2)	0.02*	1.4 (0.5–3.7)	0.52
	Peritoneal involvement	1.4 (0.5–4)	0.57			1.6 (0.7–4.1)	0.26			3.1 (1.1–8.6)	0.02*	1.2 (0.4–3.2)	0.85
	Shepherd’s 4 grade	3.6 (1–14)	0.04*	2.9 (1.1–9.5)	0.04*	2.7 (1.1–10)	0.05*	2.1 (0.7–6.7)	0.21	7.3 (1.9–27.8)	0.001*	4.2 (1.2–16.9)	0.04*
	pCRM+	4.8 (1.3–17)	0.009*	3.1 (1.1–8.8)	0.03*	8.1 (2–32)	0.001*	3.3 (1.4–8.1)	0.007*	6.1 (1.7–21.9)	0.002*	4.1 (1.1–15.5)	0.03*

Hazard ratio (95% CI)

*Statistically significant *p*-value (*p*<0.05)

Abbreviations: *PR* peritoneal reflection, *rm* magnetic resonance-preoperative staging, *T4* T4 staging on TNM classification, *CRM* circumferential resection margin, *N+* positive adenophaties, *CRT* hemoradiotherapy, *RBC* red blood cell, *TME* total mesorectal excision, *p* final pathological staging

## Discussion

This study assesses the prognostic relevance of peritoneal involvement at PR in rectal cancer and evidences that is a strong predictor of LR, with an adjusted-HR 4.2 (95% CI 1.2–16.9) for grade 4 Shepherd’s involvement. Peritoneal involvement at that location was present in 24 out of 85 (28.2%) tumors above or at the level of PR and 29.1% (7/24) of these patients developed LR, all presenting with carcinomatosis. Notably, while serosal ulceration with tumor cells free in the peritoneum (grade 4 involvement) was found in only 6.25% of all patients, this subgroup developed LR in 50% of cases. This data is in concordance with previous publications by Shepherd and colleagues and highlights the importance of the peritoneal involvement in the oncological outcomes [25, 26].

Anterior rectal tumors at the level of peritoneal reflection can, potentially, reach the peritoneal surface and/or the anterior mesorectal fascia as the mesorectal fat becomes very thin at this level [12, 30, 32]. In this regard, the European Society of Gastrointestinal and Abdominal Radiology (ESGAR) recommends to describe the relationship of rectal tumors with the anterior peritoneal reflection, as tumor’s invasion above the level of PR at the anterior side should be considered at risk for peritoneal involvement rather than anterior CRM involvement [17, 33, 34]. However, although MRI has demonstrated a high accuracy for determination of tumor’s location according to PR, preoperative evaluation of PR involvement can be challenging [24], and may not have been adequately considered in the analysis of the prognostic implications of the circumferential location of distal cancer [2].

Estimated rectal cancer carcinomatosis rate is 3–4.2% but pT4 tumors present up to 10 times higher risk of carcinomatosis [11, 13, 35, 36]. Patients with rectal cancer and resected local carcinomatosis at the primary surgery present worse median OS (48 vs. 97 months, *p* < 0.001) and a 5-year LR of 15.7% [35]. In our series, pT4 tumors accounted for 21.9% of cases, with LR developing in 28.6% of these cases: 9 patients as carcinomatosis, while 1 patient developed pelvic recurrence.

To date, guidelines recommend upfront surgery for rectal tumor above the PR but peritoneal involvement is not taken into account [22]. Marinello et al. reviewed 1145 patients comparing oncological outcomes of sigmoid and rectal tumors and concluded that upper rectal tumors can be managed as sigmoid cancer without neoadjuvant chemoradiotherapy and subtotal mesorectal excision with similar outcomes (LR 4.9% vs. 7%, *p* > 0.05) [8]. For tumors located in the upper rectum, a subtotal total mesorectal excision (TME) may be considered; however, for tumors in the mid or lower rectum, a complete TME is mandatory to ensure adequate oncological clearance. For all anterior tumors, a dissection anterior to Denonvilliers’ fascia is recommended to ensure a clear circumferential resection margin but PR involvement does not alter the standard surgical technique.

However, due to the high incidence of local recurrence as carcinomatosis when peritoneal involvement is present, existing strategies are being reevaluated to improve oncological outcomes. These have mainly focused on two strategies: neoadjuvant treatment aimed to achieve tumor’s regression and intensive follow-up for early detection and treatment of local recurrence.

Recent publications advocate for neoadjuvant systemic chemotherapy (NAC) in the management of locally advanced colon cancer. The finding of the FOxTROT trial suggest that NAC improves tumor control by reducing incomplete resections and promoting higher regression rates. The primary end point of achieving improved 2-year disease control for cT3-4 colon cancer seems to be achieved, with a HR 0.72, 95% CI 0.54–0.98; *p* = 0.037 [35]. Additionally, the more recent OPTICAL trial also compares NAC regime for cT3-4 colon cancer with upfront surgery. While this trial did not reveal superior 3-year DFS in the NAC group, it showed a potentially improved OS (HR 0.44, 95% CI 0.25–0.77) [36]. Ongoing trials, such as the ELECLA trial [37], aim to provide further evidence on NAC strategies for locally advanced colorectal tumors.

Therefore, most recent studies advocate for neoadjuvant strategies for the management of locally advanced colorectal tumors, NAC for colonic and TNT including radiotherapy for lower rectal tumors. Given this new trend, it seems logical to consider neoadjuvant treatment strategies for tumors above the PR when preoperative suspected serosal involvement is present, which have traditionally undergone direct surgical resection. It remains to be determined what the optimal neoadjuvant regimen might be considering the limitations of MRI in accurately assessing PR involvement [24, 38, 39].

On the other hand, several authors have evaluated different intensive follow-up strategies for patients at high risk of developing peritoneal carcinomatosis following surgery for colorectal tumors. Patients with PR involvement are at high risk of local recurrence as shown in this publication. Given the limitations of CT scan in the early detection of peritoneal carcinomatosis, pelvic MRI could be considered as part of an intensive follow-up protocol in this subgroup of patients. A meta-analysis of 17 trials evaluating intensive follow-up demonstrated that patients were twice as likely to undergo salvage surgery after interval recurrence; however, this approach failed to demonstrate improved cancer-related OS [40]. The COLOPEC trial evaluated the role of adjuvant oxaliplatin-based hyperthermic intraperitoneal chemotherapy (HIPEC) for pT4 or perforated colon cancer [41]. The results showed no improved peritoneal metastasis-free survival at 18 months (80.9% vs. 76.2%). Similarly, the PHROPHYLOCHIP-PRODIGE 15 trial aimed to investigate the role of prophylactic oxaliplatin-based HIPEC following primary colonic resection with synchronic local carcinomatosis removal, excision of ovarian metastases, or treatment for tumor perforation [42]. After a 5-year follow-up, they failed to demonstrate improved DFS compared to standard surveillance alone. The more recent HIPECT4 trial, assesses the efficacy of concomitant mitomycin C–based HIPEC for cT4 colon and rectal tumors above PR during primary surgery [43]. The 3-year local control (LC) rate was higher in the HIPEC group (97.6% vs. 87.6%, *p* = 0.03) but there were no differences in DFS and OS. Within the subgroup with pT4 disease (67.9% of enrolled patients), there was a pronounced benefit in 3-year LC in the HIPEC group (98.3% vs. 82.1%, *p* = 0.003; HR 0.09, 95% CI 0.01–0.70).

In view of this results, NAC strategies for intraperitoneal rectal tumors involving the peritoneal reflection could be extrapolated from NAC regimens used in trials for locally advanced colon tumors. In addition, the role of prophylactic HIPEC in T4 intraperitoneal colorectal tumors remains uncertain. Randomized control trials are needed to determine the optimal neoadjuvant and adjuvant therapies for upper rectal tumors with PR involvement as it may have major implications in oncological outcomes.

This study presents several limitations. Firstly, due to the study being conducted in a single institution with restricted patient enrolment, the sample size is limited. However, we believe that the results are of significant value due to their high methodological quality, despite the modest sample size. Furthermore, patients undergoing neoadjuvant chemoradiotherapy with PR involvement on preoperative MRI may not demonstrate serosal involvement in the pathological examination due to tumor regression. However, microscopic peritoneal spread may already exist within the abdominal cavity, potentially leading to early recurrence. Moreover, computed tomography scanners have inherent limitations in detecting peritoneal nodules smaller than 1 cm, with published accuracy ranging from 44 to 93.8% [44]. Consequently, the actual incidence of LR may be underestimated during follow-up. Moreover, as the study was conducted between 2016 and 2019, only long-course QRT neoadjuvant treatment was considered in our institution’s protocol that differs from current TNT neoadjuvant strategies. Although the strength of our study lies in the long-term follow-up, we are aware that this represents a limitation when comparing our results with more recent studies.

This study demonstrates that peritoneal reflection involvement is a strong predictor of both local recurrence and poor overall survival in rectal cancer. These findings highlight the importance for multidisciplinary colorectal teams to consider peritoneal reflection involvement when discussing treatment strategies for rectal tumors.

## Data Availability

The data that support the findings of this study are not openly available due to reasons of sensitivity and are available from the corresponding author upon reasonable request.
